# A Unique Feature of Iron Loss via Close Adhesion of *Helicobacter pylori* to Host Erythrocytes

**DOI:** 10.1371/journal.pone.0050314

**Published:** 2012-11-21

**Authors:** Zhiwei Wang, Lijuan Zhang, Zhi Guo, Lei Liu, Jun Ji, Jianian Zhang, Xuehua Chen, Bingya Liu, Jun Zhang, Qiulan Ding, Xuefeng Wang, Wei Zhao, Zhenggang Zhu, Yingyan Yu

**Affiliations:** 1 Shanghai Institute of Digestive Surgery and Department of Surgery, Shanghai Ruijin Hospital, Shanghai Key Laboratory for Gastric Neoplasia, Shanghai Jiao Tong University School of Medicine, Shanghai, China; 2 Shanghai Synchrotron Radiation Facility, Shanghai Institute of Applied Physics, Chinese Academy of Science, Shanghai, China; 3 Department of Transfusion and Clinical Biochemistry, Shanghai Ruijin Hospital, Shanghai, China; 4 Department of Medical Microbiology and Parasitology, Shanghai Jiao Tong University School of Medicine, Shanghai, China; University of Toronto, Canada

## Abstract

Iron deficiency anemia is an extra-stomach disease experienced in *H. pylori* carriers. Individuals with type A blood are more prone to suffering from *H. pylori* infection than other individuals. To clarify the molecular mechanisms underlying *H. pylori*-associated anemia, we collected erythrocytes from A, B, O, and AB blood donors and analyzed morphology, the number of erythrocytes with *H. pylori* colonies attached to them, and iron contents in erythrocytes and *H. pylori* (NCTC11637 and SS1 strains) by means of optical microscopy, scanning electron microscopy, and synchrotron radiation soft X-ray imaging. The number of type A erythrocytes with *H. pylori* attached to them was significantly higher than that of other erythrocytes (*P*<0.05). Far more iron distribution was observed in *H. pylori* bacteria using dual energy analysis near the iron *L*2, 3 edges by soft X-ray imaging. Iron content was significantly reduced in host erythrocytes after 4 hours of exposure to *H. pylori*. *H. pylori* are able to adhere more strongly to type A erythrocytes, and this is related to iron shift from the host to the bacteria. This may explain the reasons for refractory iron deficiency anemia and elevated susceptibility to *H. pylori* infection in individuals with type A blood.

## Introduction

*Helicobacter pylori* (*H. pylori*) is a Gram-negative microanaerobic bacillus, which colonized the human gastric mucosa. Persistent infection by *H. pylori* causes chronic inflammation, ulcers, and even stomach neoplasm in infected individuals. Chronic infection with *H. pylori* also leads to extradigestive diseases, such as autoimmune disease and diabetes mellitus. Iron deficiency anemia is one of the most common diseases outside the stomach, especially in children and adolescents [1], [2], [3], [4], [5], [6], [7]. A meta-analysis of epidemiological studies revealed a correlation between *H. pylori* and iron deficiency anemia (OR, 2.22; 95% CI: 1.52–3.24)[5]. *H. pylori*-related iron deficiency anemia is defined as follows: (1) Patients lack obvious gastrointestinal manifestations; (2) patients lack bleeding mucosa lesions under the endoscopic examination; (3) no evidence that iron intake is insufficient; (4) detectable *H. pylori* infection in patients with chronic gastritis; (5) poor response to iron agent supplementation; (6) dramatic response to eradication therapy targeting *H. pylori*, even when no iron agent replenishment is given, even to the point of serum iron level recovery in some patients. In general, iron deficiency anemia tends to be attributed to insufficient iron intake, chronic blood loss, malabsorption, and hemolysis. However, another important cause of iron deficiency anemia may be *H. pylori* infection.

Nakao and colleagues reported that both *H. pylori* infection and stomach disease are closely associated with genotypes of human ABO blood groups. They demonstrated that the rates of *H. pylori* infection, chronic atrophic gastritis, and gastric cancer were higher in individuals with type A blood than in other individuals [8]. As early as 1953, Aird found an increased susceptibility to gastric cancer among individuals with type A blood [9]. So far, there has been no reasonable explanation for the special clinical phenomena on a molecular level. In order to uncover the possible mechanisms of *H. pylori*-related iron deficiency anemia, and of increased risk for *H. pylori* infection in type A individuals, we collected erythrocytes from donors in A, B, O, and AB blood groups and observed interactions between those erythrocytes and *H. pylori* ex vivo using optical microscopy, scanning electron microscopy, and third-generation synchrotron radiation.

A synchrotron-based scanning transmission soft X-ray microscopy (STXM) is especially well-suited to the study of the distribution of organic *K*-edge and metal *L*-edge analysis. Previously, we used the new technique to study the imaging of biological samples and element distribution [10], [11]. In the current study, we examined the interactions between *H. pylori* and human erythrocytes from ABO blood groups and to examine the distribution of iron. This is the first report to assess interactions between human erythrocytes and *H. pylori* pathogen using optical microscopy, scanning electron microscopy, and third-generation synchrotron radiation. Our research will provide a valuable reference for study of the crucial mechanisms underlying *H. pylori*-related iron deficiency anemia.

## Materials and Methods

### Erythrocyte preparation

Erythrocytes from individuals with ABO blood types were identified using standard erythrocyte antiserum agglutination methods. A 5 ml fresh blood from healthy volunteers of A, B, O, and AB blood groups was centrifuged in anticoagulant tubes (3000 rpm for 10 min). Twenty-microliter samples of erythrocytes were washed twice with phosphate buffered saline (PBS, pH 7.4). Cells were counted on a hemacytometer before experiments.

### Ethics Statement

Written informed consent in the study was obtained from all participants. The study protocol was approved by the ethics committee of Ruijin Hospital, Shanghai Jiao Tong University School of Medicine.

### *H. pylori* preparation

*H. pylori* strains NCTC11637 and SS1 (both CagA- and VacA-positive) were provided by Professor Guo of the Department of Medical Microbiology and Parasitology, Institutes of Medical Sciences, Shanghai Jiao Tong University, School of Medicine. *H. pylori* strains were cultured routinely for 72 h on Columbia agar base (Biomerieux, France) with 5% sheep blood in mixed air containing 10% CO_2_, 5% O_2_, and 85% N_2_ at 37°C. Then, we converted *H. pylori* to liquid medium containing brain heart infusion (BD, U.S.), 10% sheep blood, and the same antibiotics as those used in Columbia agar base. The liquid medium was shaken on a shaking table (Forma Scientific, U.S.) with a constant rotation rate of 120 rpm. *H. pylori* were counted using a spectrophotometer (BioSpec-min, Shimadzu Scientific Instruments, Japan) and washed with sterile PBS (pH 7.4, 5000 rpm, 10 min) before use.

### Co-culture of erythrocytes with *H. pylori*

Four types of erythrocytes (A, B, O, and AB) were seeded into six well plates at a density of 1×10^6^ per well for each type of erythrocyte. Each type was seeded at quadruple wells. The ratio of the bacteria to erythrocytes was 100∶1. The six well plate was put into the cell culture incubator. After incubation for 4 hours, the mixture of erythrocytes and *H. pylori* was washed twice using sterile PBS (700 rpm for 5 min) and resuspended in 100 µl PBS.

### Wright-Giemsa staining

We resuspended the erythrocytes by 100 µl PBS and smeared on glass slides sterilized by exposure to ultraviolet lamplight for 4 hours. Samples were air dried and slides were fixed by methanol for 2 min. Then reagent A of Wright-Giemsa was dropped onto the slides for 1 min (Baso Diagnostic Inc, Zhuhai, China) followed by reagent B for another 5 min. The slides were washed with distilled water, air dried, sealed using neutral balsam, and covered by cover slips. Interactions between *H. pylori* and erythrocytes were observed via light microscopy (Olympus BX51, Japan). Erythrocytes with *H. pylori* colonies attached to them were counted oil immersion lens. Ten random fields were counted for each type of erythrocyte.

### Scanning electron microscopy

The mixtures of erythrocytes and *H. pylori* colonies were resuspended in 100 µl PBS and dropped on a piece of supporting slide (0.5 cm×0.5 cm) which had been sterilized by exposure to ultraviolet lamplight for 4 hours. Samples were air dried and slides were immersed in 2.5% glutaraldehyde in 0.1 M cacodylate buffer (pH 7.4) for 2 hours at room temperature. Then slides were washed 3 times (5 min for each time) in 0.1 M cacodylate buffer (pH 7.4). The slide was re-fixed in 1% osmium tetroxide (aqueous, pH 7.4) for 1 hour at room temperature in a dark container. They were then washed 3 times (5 min each) in 0.1 M cacodylate buffer (pH 7.4) and dehydrated using ethanol at different concentrations. The samples were subjected to critical point drying and mounted onto a metal stub using double-sided carbon tape. Finally, the samples were plated on a layer of metal (gold and palladium) using an automated sputter coater and observed using scanning electron microscopy (FEI, Netherlands).

### STXM experiments

STXM imaging and Fe L-edge NEXAFS measurements was performed at the beamline BL08U1A of the Shanghai Synchrotron Radiation Facility (SSRF). Mixtures of erythrocytes with *H. pylori* colonies were resuspended in 100 µl PBS and dropped on a piece of silicon nitride windows (Shanghai NTI Co. Ltd, China) which had been sterilized by exposure to ultraviolet lamplight for 4 hours. The samples were air-dried and the silicon nitride window was immersed in 2.5% glutaraldehyde in 0.1 M cacodylate buffer (pH 7.4) for 2 hours at room temperature. Then the samples were mounted onto the sample holder of the device and observed by soft X-ray spectromicroscopy. The distribution map of iron element was obtained by digital division of two absorption-contrast images at dual photon energies of *E1* = 704 eV and *E2* = 707.8 eV, which are at and just before the edge of iron absorption. Single-energy images at selected energy levels were scanned and recorded as raw data. The cluster analysis method can be used to obtain not only Fe speciation maps but also the corresponding NEXAFS spectra. First, sequences of images (“stacks”) were recorded at a series of energies around the relevant absorption edge (695–720 eV for Fe L edge). Then they were aligned via spatial cross-correlation analysis. Finally, NEXAFS spectra were extracted from groups of pixels with similar absorption features within the image region of interest using the IDL package aXis2000.

### Statistical analysis

SPSS (11.0 version) software and one-way analysis of variance (ANOVA) for erythrocyte or *H. pylori* counts in different blood groups were used. *P*<0.05 indicates significant differences.

## Results

### Wright-Giemsa histochemical staining

**Figure 1:**
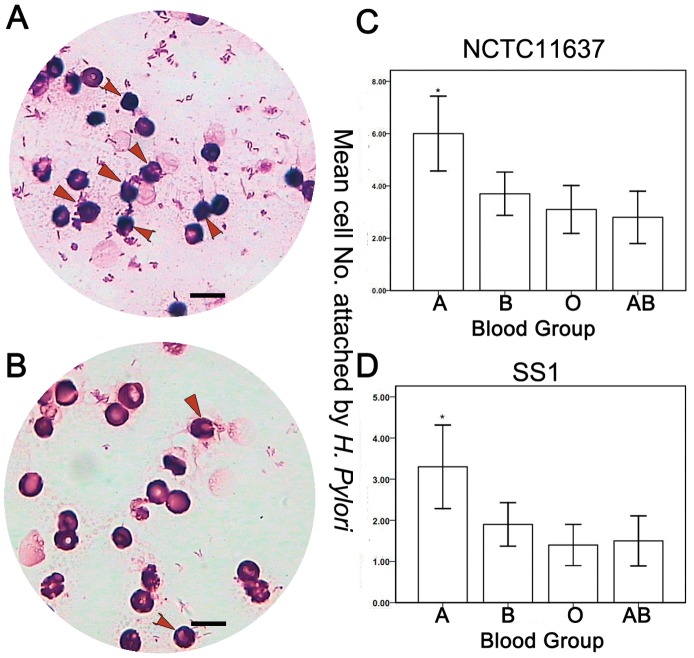
Co-culture of NCTC11637 strain with erythrocytes. (A) Type A erythrocytes. More type A erythrocytes have *H. pylori* attached to them (arrow). (B) Type O erythrocytes. These have fewer *H. pylori* attached to them (arrow). (C) The mean number of erythrocytes with *H. pylori* NCTC11637 strain in each ABO blood group is shown in the bar chart. * indicates that the number of type A erythrocytes is significantly higher than other groups (*P*<0.05). No significant difference is found between other blood groups. (D) The mean number of erythrocytes with *H. pylori* SS1 strain in each ABO blood group is shown in the bar chart. * indicates that the number of erythrocytes in blood group A was found to be significantly higher than in other groups (*P*<0.05). No significant difference is found between other groups (Wright-Giemsa, 1000×). In figure 1C and 1D, the values represent as mean ± standard deviation. The scale bar reprensents the 10 µm.

Under Wright-Giemsa staining, *H. pylori* were visible in royal blue clusters. Erythrocytes were stained magenta (Figure 1). We counted the number of erythrocytes with *H. pylori* NCTC11637 strain attached to them in different blood groups. We found the average number of affected erythrocytes to be 6.0 for type A, 3.7 for type B, 3.1 for type O, and 2.8 for type AB (*P*<0.05). The average number of erythrocytes with *H. pylori* SS1 strain attached to them was 3.3 for type A blood cells, 1.9 for type B, 1.4 for type O, and 1.5 for type AB (*P*<0.05). The number of affected erythrocytes was significantly higher among type A cells than other cells (Figure 1A to 1D). No significant difference was found between blood groups B and O (*P*>0.05); blood groups B and AB (*P*>0.05); or blood groups O and AB (*P*>0.05).

### Scanning electron microscopy

**Figure 2:**
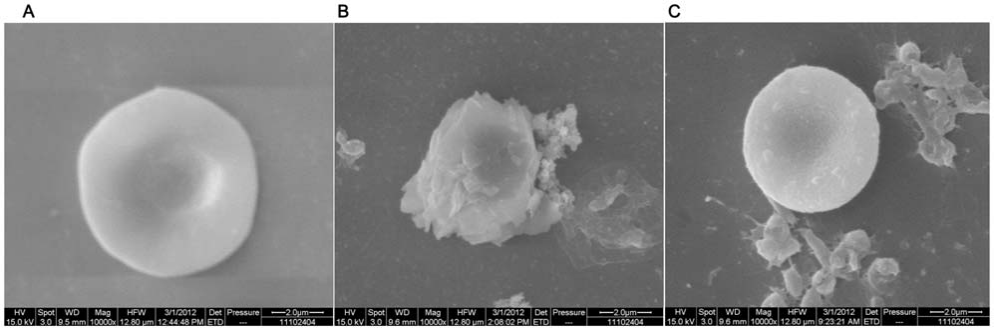
Scanning electron microscope observation of interaction between erythrocytes and *H. pylori* NCTC11637 strain. (A) The normal erythrocytes have biconcave disc shapes with smooth surfaces. (B) There are more *H. pylori* colonies closely attached to type A erythrocytes than to other erythrocytes after 4 hours of co-cultivation. The deformation of type A erythrocytes was more pronounced than that of type O erythrocytes. (C) Fewer *H. pylori* were attached to type O erythrocytes than to type A erythrocytes. Type O erythrocytes retained their original shapes. Only small protrusions appear on the surfaces of type O erythrocytes (10,000×).

Under scanning electron microscopy, normal erythrocytes appeared as biconcave disc shape with smooth surfaces and diameters of 5–7 µm (Figure 2A). After co-cultivation with *H. pylori* strains for 4 hours, small protrusions appeared on the surfaces of the erythrocytes (Figure 2B). We noticed, compared with type O erythrocyte, type A erythrocyte (Figure 2B) appeared swelling and rough with more *H. pylori* colonies attachment. The deformation of type A erythrocytes was more pronounced than that of type O erythrocytes(Figure 2C).

### Soft X-ray spectromicroscopy at SSRF

**Figure 3:**
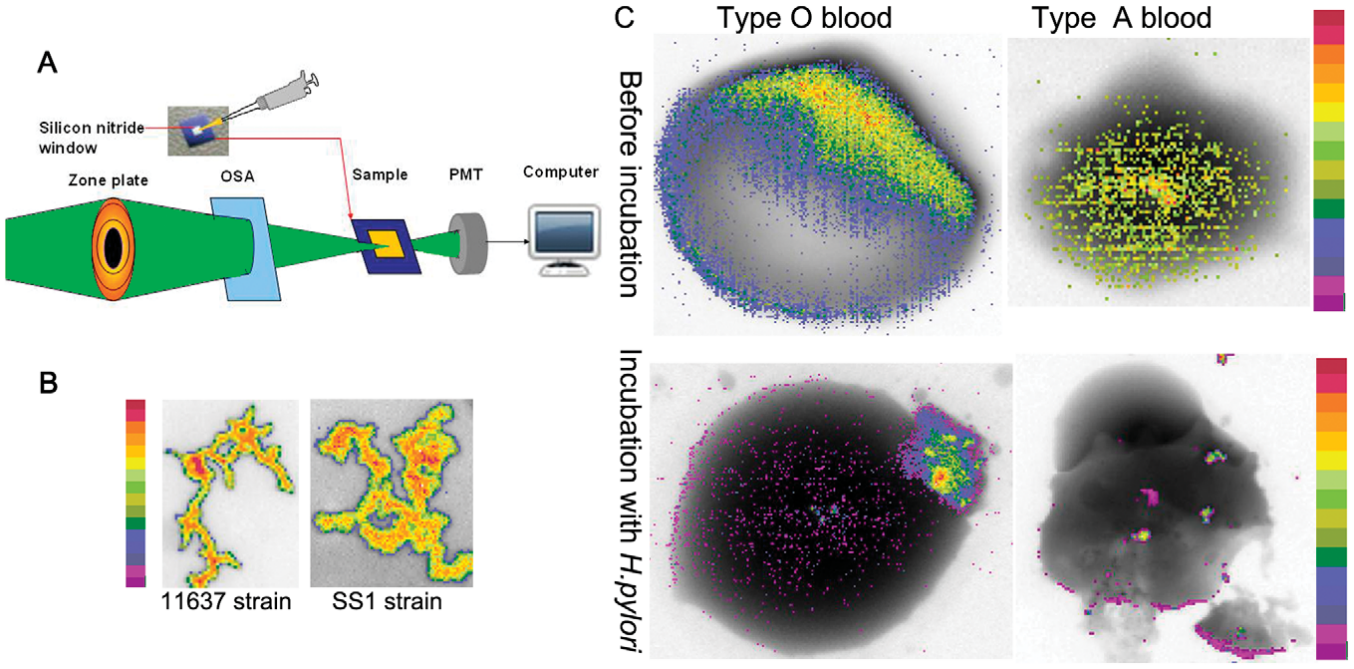
Observation of erythrocyte and *H. pylori* NCTC11637 strain by synchrotron radiation soft X-ray spectromicroscopy. (A) Schematic layout of the BL08U beamline. OSA is the order-sorting-aperture. PMT is photomultiplier tube. (B) Dual-energy (707.8 eV and 704 eV) contrast images of *H. pylori* and iron element. Colored bar on the left side indicates the iron content. From bottom to top, the gradient colors indicate increased iron content. The NCTC11637 strain is shown on the left and the SS1 strain is shown on the right. Two strains appear orange-yellow or light yellow, which means that these *H. pylori* contained more iron than nearby erythrocytes. (C) Dual-energy (707.8 eV and 704 eV) contrast images of erythrocytes with the iron element. Colored bars on the right side indicate iron content. Type O erythrocyte is on the left and type A erythrocyte is on the right. The color bar represents relative content changes. It has 16 different colors from bottom to top, which correspond the iron element content. For instance, the purple represents lower iron content, and the orange represents higher iron content. From Figure 3C we noticed, before incubation with *H. pylori,* the iron content is rather high (orange to yellow). However, after incubation with *H. pylori,* the iron content of erythrocyte is obvious low (purple). Part of the cell membrane of the type A erythrocyte in the right lower corner is damaged.

**Figure 4:**
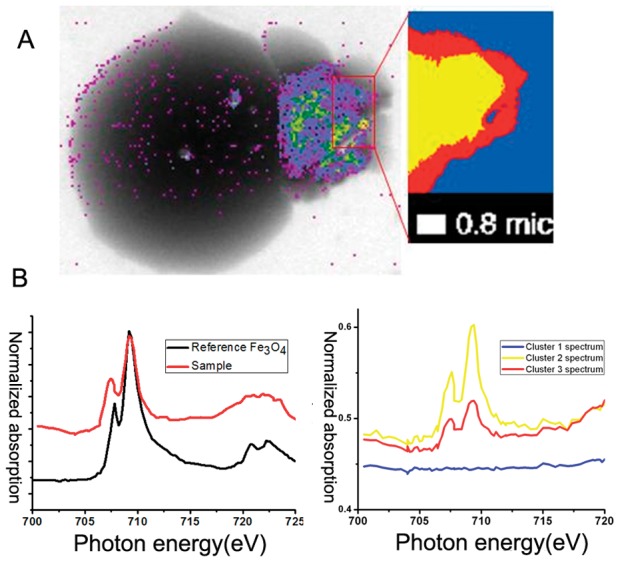
Stack analysis of the interaction between human erythrocyte with NCTC11637 strain. (A) We selected a portion of the bacterial colonies (magnification on upper-right corner) for the stack scan. (B) Left: the image of photon absorption was obtained from this stack scan for standard Fe_3_O_4_. Right: the image of photon absorption was obtained from this stack scan for NCTC11637 strain. The horizontal labels indicate photon energy and the vertical axis indicates normalized absorption. The yellow, red, and blue lines corresponded to the color regions of the figure (A, magnification on upper-right corner) using *L*-edge soft X-ray imaging.

The structure and iron distribution of erythrocytes after exposure to *H. pylori* were analyzed by soft X-ray spectromicroscopy at BL08U1A beamline of SSRF (Figure 3A). The beam is focused by a zone plate to the sample with a beam size greater than 30 nm. We imaged erythrocytes that had been exposed to *H. pylori* at *E1* = 704 eV and *E2* = 707.8 eV, which are just below and at the absorption edge of iron. These two images were analyzed using dual-energy contrast analysis. Figure 3B shows absorption-contrast images of NCTC11637 and SS1 strains of *H. pylori*. Figure 3C show typical absorption-contrast images of human type O and type A erythrocytes at two photon energies. In Figure 3, the color bar disclosed 16 different color from bottom to top, which corresponds the iron element content. For instance, the purple represents lower iron content, and the orange represents higher iron content. From Figure 3C we noticed, before incubation with *H. pylori,* the iron content of erythrocyte is rather high (orange to yellow). However, after incubation with *H. pylori,* the iron content of erythrocyte is obviously low (purple). The color bar represents the relative content changes. The reduction of iron content in type A erythrocytes is more pronounced than in type O erythrocytes. Type A erythrocytes were also found to rupture (Figure 3C, down). We used a stack scan to analyze the chemical valence of iron in *H. pylori* colonies and found the iron inside bacteria to be mainly ferric iron, as compared to standard sample of Fe_3_O_4_, and the concentration of iron inside bacteria is higher than in the bacterial membrane (Figure 4).

## Discussion

*H. pylori* is a pathogen closely related to chronic gastritis, ulcers, and stomach cancer. It has been defined as a class I carcinogen by the World Health Organization. The risk of gastric cancer is significantly reduced after eradication therapy of *H. pylori* [12]. *H. pylori* is divided into two different phenotypes, strains that contain vacuolating toxin (VacA) and cytotoxin-associated gene (CagA), and strains that do not. The adhesion and colonization of *H. pylori* on host cells is a basic characteristic of the species. The outer membrane proteins play an important role in adhesion and colonization. More than 30 outer membrane proteins have been identified on *H. pylori* [13]. Adhesion to host cells is attributable to proteins such as blood group antigen binding adhesin A and B (BabA, BabB), hop-related proteins B (HorB) and sialic acid-binding adhesin (SabA). [14]. BabA mediates bacterial adherence to human blood group antigens in the gastric epithelium. It is encoded by the BabA2 gene [15]. The BabA2-positive strain is correlated with the activity of gastritis in the antrum and corpus. Alterations in this gene have been found to have severe effects. Adherence of *H. pylori* via BabA appears to be of importance to efficient delivery of VacA and CagA and may play a role in the pathogenesis of severe histological changes associated with gastric lesions, including intestinal metaplasia and glandular epithelial atrophy [16]. Yu and colleagues analyzed the association between the BabA2 gene and chronic gastritis in 104 *H. pylori* carriers and noted that increased epithelial proliferation in individuals infected with BabA2 (+) strains. The presence of BabA2 (+) *H. pylori* strains alone or in combination with CagA (+) and VacA (+) was associated with the presence of precancerous lesions [17].

Over the last few decades, several studies have shown that the individuals with *H. pylori* infection always experience unexplained, even refractory iron deficiency anemia [2], [3], [18], [19]. The possible causes of this phenomenon include the following: (1) The bacteria cause chronic gastrointestinal bleeding and decrease iron storage. However, bleeding lesions are rarely observed under the endoscopic examination, and fecal occult blood tests are usually negative. This does not support the conclusion that chronic bleeding is the cause of *H. pylori*-related iron deficiency anemia [20], [21], [22], [23], [24]. (2) Atrophic gastritis induced by *H. pylori* reduces HCl secretion and interferes with iron absorption. In human beings, the duodenum and jejunum are major areas of iron absorption. Two forms of dietary iron exist in nature, heme-iron, which is found in meat, and non-heme-iron, which is found in vegetables and grains. The former is more easily absorbed than the latter. HCl plays a role in the dissolution and absorption of non-heme-iron. Acid can convert ferric iron to ferrous iron to facilitate transport through the cell membrane [25]. (3) The ascorbic acid in the gastric juices may promote the absorption of iron. However, the level of ascorbic acid in *H. pylori* carriers is much lower than in uninfected individuals [26].

**Figure 5:**
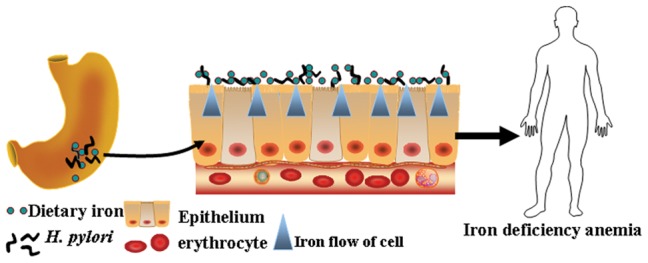
Diagram of the novel mechanism for iron deficiency anemia caused by *H. pylori.* In case of *H.Pylori* infection, the demand for iron element of *Helicobacter* is increased. *H.Pylori* colonized in stomach competitively absorbs iron with host. The dietary iron is continuously absorbed by *H.Pylori.* At the same time, the iron within the host epithelial cells is flowed out and absorbed by *H.Pyloris* gathered on the surface of epithelial, leading to iron insufficient intake in host.

This study explored the interactions between *H. pylori* and erythrocytes from individuals with different ABO blood types from multiple energy perspectives. Light microscopy, scanning electron microscopy, synchrotron radiation soft-x-ray were all used to scan the distribution and content of iron element both in bacteria and host cells. We first found that *H. pylori* had different binding capacities for host erythrocytes with different ABO blood antigens. *H. pylori* were able to bind to type A cells much more readily than to other cells. It was significantly more difficult for them to bind to type O cells than to other cells. *H. pylori* became closely attached to the erythrocyte membranes, causing significant cellular deformation. Erythrocytes co-cultivated with *H. pylori* for 4 hours also showed significant iron loss. We here propose a new probable mechanism of *H. pylori*–related anemia: the tight adhesion of *H. pylori* to host cell membrane facilitates the transfer of iron from the host cell to the bacterium (depicted in Figure 5). Optical microscopy, scanning electron microscopy, and synchrotron radiation analysis suggested that host-*H. pylori* interaction is most intense when the host cells were type A erythrocytes. This is why cell damage is more serious in individuals with type A blood than in other individuals. These findings provide a valuable explanation of the higher incidence of iron-deficiency anemia and *H. pylori* infection in individuals with type A blood than in other individuals.

X-ray spectroscopy of synchrotron radiation is a non-invasive means of analyzing biological samples. Analysis is relatively simple and does not require complex pretreatment procedures. It is suitable for analysis of multiple elements. It has already been used in the initial analysis of many biological samples, including botanical samples [11], [27], [28]. To date, there has been no report of any analysis of human red blood cells and *H. pylori* at a synchrotron radiation facility. This study is the first to explore the interaction and dynamics of the interplay between the iron element of human red blood cells after co-culture with *H. pylori* by means of the synchrotron radiation examination in a high-energy physics field. We established a double contrast method of image analysis of biological samples and identified the element using absorption edges at or below the previous energy level (*E1* and *E2*, and *E1>E2*) [11]. In this study, we demonstrated that higher concentrations of iron were present in bacteria than in human erythrocytes. This suggests that iron is an important nutrient for *H. pylori*. The stack scan analysis of the synchrotron radiation showed that ferric iron is the predominant form of iron in *H. pylori*, but there is also some ferrous iron. In the human body, ferrous iron is involved in the Fenton reaction, in which iron catalyzes hydrogen peroxide and superoxide to form hydroxyl radicals. Hydroxyl free radicals can injure cells through lipid peroxidation [29], [30], [31]. *H. pylori* mediate iron loss via close adhesion on host cell membrane. It forms a microenvironment rich in free iron ions, and then catalyzes the formation of oxygen free radicals through the Fenton reaction. Bacteria are thereby rendered resistant to oxidative stress [32], [33], [34], [35], [36]. Iron is not only an important component of proteins and enzymes in living organisms. It is also required for bacterial survival and virulence. Proteins related to iron metabolism have been identified as important factors in *H. pylori* virulence [37]. The drift of iron from host cells to *H. pylori* may explain the mechanism underlying refractory anemia in *H. pylori-*infected individuals. We also provide a valuable clue for developing strategies toward *H. pylori* intervention and eradication.

In conclusion, this study is the first to demonstrate that increased susceptibility to *H. pylori* infection is associated with the host's genetic traits, specifically ABO blood group. The ability of *H. pylori* to adhere to type A blood cells is stronger than its ability to bind to cells from individuals with other blood types. Synchrotron radiation soft X-ray spectromicroscopy was used to explore the molecular mechanism of *H. pylori-*related iron deficiency anemia. Our findings may be helpful to interpret the unexplained iron deficiency anemia in *H. pylori* infections and support the conclusion that type A individuals are at higher risk of *H. pylori* infection.
